# Efficacy and Safety of Mepolizumab in the Management of Severe Eosinophilic Asthma: A Systematic Review

**DOI:** 10.7759/cureus.49781

**Published:** 2023-12-01

**Authors:** Ibrahim M Dighriri, Anas I Alnughaythir, Amna A Albesisi, ‏Danya I Alhuwaimel, Alanoud S Alotaibi, Laila A Alghowaidi, Fatimah H Almalki, Jasmine N Al-Bukhari, Tahani R Alshammari, Fahad H Alwathnani, Abdulmohsen A Alghamdi, Ali A Alghamdi, Safar D Alshehri, Nora Y Mahnashi, Hassan A Abu Jamilah

**Affiliations:** 1 Department of Pharmacy, King Abdulaziz Specialist Hospital, Taif, SAU; 2 Department of Pharmacy, Security Forces Hospital, Riyadh, SAU; 3 Department of Pharmacy, East Jeddah Hospital, Jeddah, SAU; 4 Department of Pharmacy, Alrass General Hospital, Alrass, SAU; 5 Department of Pharmacy, United Pharmacy, Taif, SAU; 6 ‏Faculty of Pharmacy, Taif University, Taif, SAU; 7 Department of Pharmacy, Jazan General Hospital, Jazan, SAU; 8 Faculty of Pharmacy, Taif University, Taif, SAU; 9 Department of Pharmacy, King Salman Specialist Hospital, Hail, SAU; 10 Department of Pharmacy, Prince Mishari Bin Saud Hospital, Baljurashi, SAU; 11 Department of Pharmacy, King Abdullah Hospital, Bisha, SAU; 12 Department of Community Pharmacy, Al-Amal Hospital, Jazan, SAU; 13 Pharmaceutical Care Administration, Sharurah Armed Forces Hospital, Sharurah, SAU

**Keywords:** sever asthma, safety, efficacy, mepolizumab, eosinophilic asthma

## Abstract

Severe eosinophilic asthma (SEA) is characterized by persistent airway inflammation and frequent exacerbations despite standard treatments. Mepolizumab, a monoclonal antibody that reduces eosinophil levels by targeting interleukin-5, has emerged as an add-on therapy for patients with SEA. This systematic review evaluated mepolizumab's efficacy and safety for treating SEA. A comprehensive literature search was conducted across major databases. Thirty-two studies with over 6,000 patients were included, comprising randomized controlled trials, open-label extensions, and real-world observational analyses. Study quality and risk of bias were assessed using standard tools. Meta-analysis was deemed inappropriate due to heterogeneity. Instead, a narrative synthesis was performed. Mepolizumab significantly reduced exacerbation rates by around 50% and improved symptoms and lung function compared to placebo in pivotal trials. Long-term open-label studies showed sustained reductions in exacerbations and stable lung function for up to 4.5 years. Real-world data demonstrated consistent 50%-90% exacerbation decreases across diverse patient populations over 6-24 months. Mepolizumab exhibited an acceptable safety profile, with mild injection site reactions and headaches as most common adverse events. While specific subgroups may show enhanced responses, mepolizumab displayed broad efficacy regardless of patient demographics or phenotypes. The extensive evidence provides robust support for mepolizumab as an efficacious and safe add-on treatment option for patients with severe, refractory eosinophilic asthma. Further high-quality comparative effectiveness research is warranted to optimize patient selection and positioning among emerging biologics.

## Introduction and background

Severe eosinophilic asthma (SEA) is a chronic respiratory condition characterized by ongoing airway inflammation and heightened levels of eosinophils [1]. This inflammation results from eosinophils, a type of white blood cell that plays a significant role in airway inflammation across various diseases, including allergic and non-allergic asthma, chronic rhinosinusitis, and chronic obstructive pulmonary disease (COPD) [2,3]. SEA is marked by elevated eosinophil counts in blood and sputum and airway inflammation, which can lead to airway obstruction caused by mucus plugs, frequent exacerbations, declining lung function, and even fatalities [4]. It constitutes the most common type of asthma, accounting for approximately 84% of all asthma cases and 50% of severe asthma cases [5,6]. Traditional treatments, like high-dose oral corticosteroids (OCSs), may not be effective for certain patients [4,7].

Mepolizumab is a humanized monoclonal antibody that targets interleukin-5 (IL-5), a cytokine that regulates eosinophil development and differentiation [8,9]. By reducing eosinophil levels, mepolizumab can enhance asthma control and reduce patient exacerbation [10]. It has gained approval as an additional therapy for individuals with SEA [8,9]. Multiple real-world studies have investigated the efficacy of mepolizumab for individuals who have SEA [11-13]. These studies consistently indicate significant improvements in clinical outcomes resulting from mepolizumab treatment. A long-term, multi-center study demonstrated that mepolizumab continued to reduce exacerbation and OCS usage in patients [11]. The COSMEX (COSMOS Extension) study provided evidence of mepolizumab's long-term safety and effectiveness, reducing exacerbation rates while improving asthma control and lung function [12]. Moreover, a 3.5-year trial focusing on mepolizumab's long-term safety and effectiveness produced positive results, including lower exacerbation rates and better asthma control [13].

Past studies have shown that mepolizumab, benralizumab, dupilumab, and tezepelumab all successfully reduce exacerbation rates and enhance lung function in individuals with SEA [14]. Given the challenges faced by individuals with SEA, including poorly controlled asthma and frequent exacerbations, it is essential to evaluate mepolizumab's potential to address these issues. Consequently, this systematic review aims to assess efficacy and safety of mepolizumab based on published studies in treating SEA. A comprehensive summary of mepolizumab's clinical utility could be used to inform treatment guidelines and in decision-making situations.

## Review

Methods

Search Strategy and Selection Criteria

A systematic search was conducted in PubMed, Embase, the Cochrane Central Register, the Web of Science, and Google Scholar. The entire search strategy included the terms "mepolizumab," "anti-IL-5", "anti-interleukin 5", "eosinophilic asthma," "severe asthma," "exacerbations," and related synonyms combined by using Boolean operators. The search was limited to human studies published in English. Reference lists of all included studies and relevant reviews were screened.

Randomized controlled trials (RCTs), quasi-RCTs, non-RCTs, and prospective and retrospective cohort studies were eligible for inclusion. Eligible participants included adults and children (older than six years) with SEA, defined as a blood eosinophil count ≥300 cells/μL and/or sputum eosinophilia ≥3%. Mepolizumab, administered via intravenous or subcutaneous (SC) route, was the intervention of interest. Studies were required to evaluate mepolizumab with a parallel control (placebo or active comparator) or report pre- and post-treatment data. The primary outcomes were the annual exacerbation rate and adverse events. Secondary efficacy outcomes included lung function like forced expiratory volume in the first second (FEV1), Asthma Control Questionnaire (ACQ) score, asthma quality of life questionnaire (AQLQ) score, and blood/sputum eosinophil counts.

Two reviewers performed screening independently, first at the title/abstract level, followed by a full-text review. Any disagreements were resolved through consensus or consultation with a third reviewer. Non-English studies, conference abstracts, case reports, and duplicate publications were excluded.

Data Extraction and Quality Assessment

A standardized data extraction form was used to collect relevant information from each study, including study details (author, year, design), population characteristics, intervention details (dose, frequency, duration), comparators, and outcomes. The risk of bias was assessed using Cochrane Risk of Bias tool for RCTs and the Newcastle-Ottawa scale for observational studies. Two independent reviewers carried out quality assessments, with disagreements resolved by consensus.

Data Synthesis

The search results were imported into EndNote for deduplication. Two reviewers then reviewed titles and abstracts for potential relevance. Full texts of potentially eligible studies were retrieved and assessed for inclusion based on predefined criteria. A narrative synthesis was conducted due to heterogeneity of study designs and outcome measures. A meta-analysis was deemed inappropriate. Study designs were grouped into RCTs, prospective cohorts, and retrospective analyses. Within each group, results were synthesized by outcome. Exacerbation rates, lung function, symptom scores, quality of life (QoL), and safety events were summarized in all studies.

Results

Procedures for Detecting, Reviewing, and Integrating Studies

**Figure 1:**
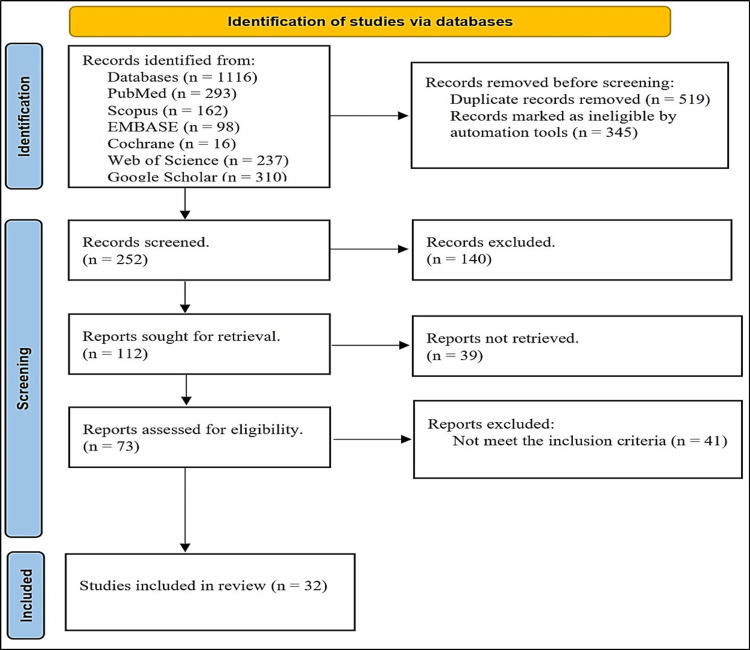
Method of detecting, reviewing, and integrating studies for this analysis

Initially, a total of 1,116 records were identified across various databases, including PubMed (293), Scopus (162), Embase (98), Cochrane (16), Web of Science (237), and Google Scholar (310). Before screening, 519 duplicate records were removed, along with 345 records deemed ineligible by automation tools, leaving 252 records for screening. Of these, 140 were excluded, and 112 reports were sought for retrieval. However, 39 reports were not retrieved, and 73 were assessed for eligibility. The eligibility assessment led to exclusion of 41 reports for not meeting inclusion criteria, culminating in 32 studies being included in this review (Figure 1).

Quality Assessment of Included Studies

Our analysis evaluated risk of bias across 32 studies, as shown in Table 1. We assessed each study for selection, performance, detection, attrition, reporting, and other potential biases, culminating in an overall risk of bias determination. The assessment of biases in the 32 included studies showed variable risks of bias. Studies that were rated as having a low risk of bias were those by Khatri et al. [13], Gunsoy et al. [15], Moore et al. [16], Kim et al. [17], Gupta, Ikeda et al. [18], Gupta et al. [19] and Yancey et al. [20]. Studies by Khatri et al. and Gunsoy et al. had low risks of bias across all domains assessed [13,15]. These studies demonstrated strengths in their selection methods, measurement techniques, reporting, and control of confounders. Fifteen studies that were rated as having an overall high risk of bias were those by Korn et al. [21], Ribas et al. [22], Harrison et al. [23], Numata et al. [24], Casale et al. [25], Yılmaz et al. [26], Taillé et al. [27], Crimi et al. [28], Llanos et al. [29], Maglio et al. [30], Atayık et al. [31], Silver et al. [32], Koistinen et al. [33], Farah et al. [34] and González-Pérez et al. [35]. The studies that had a high risk of selection bias, performance bias, and other biases included those by Ribas et al. [22], Maglio et al. [30], and González-Pérez et al. [35]. Issues included lack of randomization, lack of blinding, and a failure to control essential confounders. Four studies by Lugogo et al. [11], Khurana et al. [12], Shimoda et al. [36], and Harvey et al. [37] were rated as having an overall moderate risk of bias. These studies had some concerns with performance bias related to blinding but were otherwise low-risk.

**Table 1:** Assessment of biases in the 32 included studies

Study	Selection bias	Performance bias	Detection bias	Attrition bias	Reporting bias	Other bias	Overall risk of bias
Khatri et al. [13]	Low	Low	Low	Low	Low	Low	Low
Lugogo et al. [11]	Low	High	Low	Low	Low	Low	Moderate
Khurana et al. [12]	Low	High	Low	Low	Low	Low	Moderate
Korn et al. [21]	High	High	Low	Low	Low	High	High
Moore et al. [16]	Low	High	Low	Low	Low	Low	Moderate
Shimoda et al. [36]	Low	High	Low	Low	Low	Low	Moderate
Harvey et al. [37]	Low	High	Low	Low	Low	Low	Moderate
Ribas et al. [22]	High	High	Low	Low	Low	High	High
Harrison et al. [23]	High	High	Low	Low	Low	High	High
Kim et al. [17]	Low	High	Low	Low	Low	Low	Moderate
Numata et al. [24]	High	High	Low	Low	Low	High	High
Gupta, Ikeda et al. [18]	Low	High	Low	Low	Low	Low	Moderate
Casale et al. [25]	High	High	Low	Low	Low	High	High
Yılmaz et al. [26]	High	High	Low	Low	Low	High	High
Taillé et al. [27]	High	High	Low	Low	Low	High	High
Crimi et al. [28]	High	High	Low	Low	Low	High	High
Llanos et al. [29]	High	High	Low	Low	Low	High	High
Liu et al. [38]	Low	High	Low	Low	Low	Low	Moderate
Maglio et al. [30]	High	High	Low	Low	Low	High	High
Atayık et al. [31]	High	High	Low	Low	Low	High	High
Yancey et al. [20]	Low	High	Low	Low	Low	Low	Moderate
Gunsoy et al. [15]	Low	Low	Low	Low	Low	Low	Low
Silver et al. [32]	High	High	Low	Low	Low	High	High
Koistinen et al. [33]	High	High	Low	Low	Low	High	High
Farah et al. [34]	High	High	Low	Low	Low	High	High
González-Pérez et al. [35]	High	High	Low	Low	Low	High	High
Nagase et al. [39]	High	High	Low	Low	Low	High	High
Gupta et al. [19]	Low	High	Low	Low	Low	Low	Moderate
Kroes et al. [40]	High	High	Low	Low	Low	High	High
Loli-Ausejo et al. [41]	High	High	Low	Low	Low	High	High
Kurosawa et al. [42]	High	High	Low	Low	Low	High	High
Carpagnano et al. [43]	High	High	Low	Low	Low	High	High

Study Characteristics Across the 32 Studies

The evidence base includes a range of study designs across multiple countries globally. Pivotal RCTs like DREAM (Dose Ranging Efficacy and Safety With Mepolizumab in Severe Asthma) and MENSA (Mepolizumab as Adjunctive Therapy in Patients With Severe Asthma) initially established efficacy and safety over 32-52 weeks. Open-label extensions of these RCTs, such as COSMOS, then provided longer term data up for to 4.5 years. Real-world observational studies, including retrospective analyses and prospective cohorts, demonstrated effectiveness in broader patient populations and practice settings. The studies enrolled thousands of patients internationally, ranging in ages from 6 to over 60 years. Most evaluated 100 mg of subcutaneous mepolizumab every four weeks. Study durations ranged from 12 weeks in pediatric trials to over four years in open-label studies (Table 2).

**Table 2:** Study characteristics, efficacy, and safety of mepolizumab in 32 studies F, female; M, male; SC, subcutaneous; ACQ-5, Asthma Control Questionnaire, five-item version; OCS, oral corticosteroid; FEV1, forced expiratory volume in the first second; IV, intravenous; ACT, asthma control test; IL-5, interleukin-5; PK, pharmacokinetics; PD, pharmacodynamics; RCT, randomized controlled trial; QoL, quality of life; FeNO, fractional exhaled nitric oxide; FEF, forced expiratory flow

Study, publication year	Study design	Country	Participants	Dose	Duration	Main findings	Conclusions
Khatri et al., 2019 [13]	Open-label, single-arm, multicenter extension study	13 countries	347 (224 F, 123 M); mean age 52 years	100 mg mepolizumab SC every 4 weeks	Up to 4.5 years (mean 3.5 years)	No new safety concerns were identified with long-term treatment. The exacerbation rate was reduced by 56% during weeks 0-156 vs. off-treatment period. The ACQ-5 score improved by 0.47 points at the first assessment. Blood eosinophils were decreased by 78% at the initial evaluation; 8% anti-drug antibodies; all samples were negative for neutralizing antibodies.	Mepolizumab showed sustained efficacy and a favorable safety profile with long-term use in patients with SEA. Findings supported the use of mepolizumab as a long-term treatment option.
Lugogo et al., 2016 [11]	Open-label, multicenter, phase IIIb study	19 countries	651 (360 F, 291 M); mean age 51 years	100 mg mepolizumab SC every 4 weeks	52 weeks	Favorable long-term safety profile consistent with previous studies; low prevalence of systemic reactions (2%) and injection site reactions (4%); sustained reductions in exacerbations and OCS dose with mepolizumab; improvements in exacerbations, OCS dose, ACQ-5, FEV1, and eosinophils in patients who switched from placebo to mepolizumab	Mepolizumab showed favorable long-term safety profile and durable, stable efficacy effects in patients with SEA over 52 weeks. Findings support long-term treatment.
Khurana et al., 2019 [12]	Open-label, multicenter, long-term, phase IIIb study	18 countries	339 (161 F, 178 M); mean age 53 years	100 mg mepolizumab SC every 4 weeks	Median 2.2 years (up to 4.5 years)	No new safety signals with prolonged mepolizumab treatment; exacerbation rate: 0.93/year. Sustained improvements in ACQ-5 and FEV1; maintained reduced OCS use	Long-term mepolizumab was well tolerated and provided sustained clinical benefits in patients with SEA.
Korn et al., 2023 [21]	Retrospective analysis of registry data	Germany	Cohort 1: 131 (54 F, 77 M); mean age 55 years. Cohort 2: 220 (128 F, 92 M); mean age 56 years	100 mg mepolizumab SC	Cohort 1: 4 months. Cohort 2: ≥12 months	Clinically relevant reduction in blood eosinophils, OCS use, and improvement in asthma control with mepolizumab. Asthma control and lung function were stable after another year of mepolizumab treatment.	Mepolizumab is effective in a real-world setting, with treatment benefits maintained over time. Results are consistent with RCTs.
Moore et al., 2022 [16]	Randomized, double-blind, placebo-controlled, parallel-group study	Multiple countries	Stopped: 151 (65 F, 86 M); mean age 56 years. Continued: 144 (87 F, 57 M); mean age 57 years	Stopped: switched to placebo. Continued: 100 mg mepolizumab SC	52 weeks	Shorter time to exacerbation and loss of control when stopped mepolizumab vs. continued. Blood eosinophils increased when mepolizumab was stopped. Sustained benefits for those continuing mepolizumab.	Stopping long-term mepolizumab leads to increased exacerbations and reduced asthma control. Findings support continued treatment for sustained benefits.
Shimoda et al., 2017 [36]	Post-hoc analysis of phase III randomized, double-blind, placebo-controlled, double-dummy trial (MENSA)	Japan	Placebo: 16, mepolizumab IV: 17, mepolizumab SC: 17; mean age 55 years	75 mg IV, 100 mg SC, or placebo every 4 weeks	32 weeks	Reduced exacerbations by 90% (IV) and 62% (SC) vs. placebo. Improved morning peak expiratory flow, ACQ-5 score, and St. George's Respiratory Questionnaire score. Reduced blood eosinophils.	Mepolizumab proved to be effective and was well received by Japanese patients, with similar responses to the overall MENSA population.
Harvey et al., 2020 [37]	Observational post-marketing registry	Australia	309 (131 M, 178 F); median age 60 years	100 mg mepolizumab SC	12 months	Reduced exacerbations and hospitalizations. Improved symptom control, QoL, and lung function. Higher blood eosinophils and later asthma onset predicted a better response.	Mepolizumab is highly effective in real-world SEA, with superior responses compared to RCTs when targeted appropriately. Identified characteristics of super-responders.
Ribas et al., 2021 [22]	Multicenter observational cohort study	Spain	318 (98 M, 220 F); mean age 56.6 years	100 mg mepolizumab SC	12 months	Reduced exacerbations by 77.5%. Improved lung function and ACT score; 47.8% discontinued OCS.	Mepolizumab was effective and well-tolerated in real-world SEA, with benefits across eosinophil subgroups.
Harrison et al., 2020 [23]	Prospective, observational cohort study	7 countries	368 (142 M, 226 F); mean age 53 years	100 mg mepolizumab SC	12 months	Reduced exacerbations by 69%; reduced median OCS dose; well tolerated.	Mepolizumab demonstrated effectiveness and safety consistent with clinical trials in real-world severe asthma patients.
Kim et al., 2021 [17]	Post-hoc analysis of phase III randomized placebo-controlled trials (DREAM, MENSA)	Korea	DREAM: 24, MENSA: 45; mean age 50-52 years	75 mg IV, 100 mg SC, or placebo every 4 weeks	DREAM: 52 weeks, MENSA: 32 weeks	Reduced exacerbations with 75 mg IV and 100 mg SC vs. placebo; numerically improved lung function and symptom scores; consistent safety profile.	Mepolizumab provided clinical benefits and was well-tolerated in Korean patients with SEA.
Numata et al., 2019 [24]	Retrospective study	Japan	28 (8 M, 20 F); mean age 56 years	100 mg mepolizumab SC	Median 11 months	Improved symptoms, OCS reduction, and exacerbations; greater benefits for patients with eosinophilic chronic rhinosinusitis.	Mepolizumab provided greater benefits in severe asthma patients with eosinophilic chronic rhinosinusitis comorbidity.
Gupta, Ikeda et al., 2019 [18]	Open-label, uncontrolled, repeat-dose extension study	Multiple countries	30 (20 M, 10 F); mean age 8.6 years	40 or 100 mg mepolizumab SC based on weight	52 weeks	Well tolerated with no new safety signal; reduced blood eosinophils; reduced exacerbations.	Mepolizumab had acceptable long-term safety and efficacy in children aged 6–11 with SEA.
Casale et al., 2021 [25]	Retrospective database analysis	USA	639; mean age 50-57 years	Nor reported	12 months before and after mepolizumab initiation	Reduced exacerbations and OCS use; lower healthcare resource utilization.	Mepolizumab provided real-world benefits across comorbidities in severe asthma.
Yılmaz et al., 2021 [26]	Retrospective study	Turkey	41 (9 M, 32 F); mean age 48.8 years	100 mg mepolizumab SC	52 weeks	Reduced exacerbations and OCS use; improved symptom scores; no change in lung function.	Mepolizumab was effective in real-world SEA except for lung function.
Taillé et al., 2020 [27]	Retrospective observational study	France	146 patients; mean age 58 years	100 mg mepolizumab SC	Mean 24 months	Reduced exacerbations and OCS use; improved lung function and control.	Mepolizumab showed real-world benefits in SEA.
Crimi et al., 2020 [28]	Retrospective study	Italy	31 (13 M, 18 F); mean age 52 years	100 mg mepolizumab SC	12 months	Reduced exacerbations and OCS; improved lung function and control; no impact of comorbidities.	Mepolizumab was effective in real-world SEA, regardless of comorbidities.
Llanos et al., 2020 [29]	Retrospective database study	USA	346 patients; mean age 49 years	Not reported	12 months before and after mepolizumab initiation	Reduced exacerbations and costs; less medication use.	Mepolizumab was effective in treating real-world severe asthma.
Liu et al., 2022 [38]	Randomized, double-blind, placebo-controlled study	Multiple countries	295 patients; mean age 56 years	100 mg mepolizumab SC or placebo	52 weeks	Worse asthma outcomes after stopping mepolizumab; continued mepolizumab maintenance improvements.	Stopping long-term mepolizumab led to loss of asthma control in SEA.
Maglio et al., 2021 [30]	Retrospective study	Italy	105 (38 M, 67 F); mean age 59 years	100 mg mepolizumab SC	6-18 months	Improved FEF 25%-75%; correlated with clinical improvements.	Mepolizumab improved small airway function in real-world SEA.
Atayık et al., 2022 [31]	Retrospective study	Turkey	57 (19 M, 38 F); mean age 45 years	100 mg mepolizumab SC	≥12 months	Increased FEV1 and ACT; reduced exacerbations and OCS use.	Mepolizumab was effective in severe asthma, but there was no difference between super-responders and responders.
Yancey et al., 2019 [20]	Pooled analysis of clinical trials	Multiple countries	34 patients; mean age 15 years	75-750 mg IV or 100 mg SC mepolizumab	Up to 52 weeks	Reduced exacerbations; consistent efficacy and safety.	Mepolizumab had comparable efficacy and safety in adolescents and adults with SEA.
Gunsoy et al., 2018 [15]	Pooled analysis of clinical trials	Multiple countries	1192 patients; mean age 50 years	75 mg IV or 100 mg SC mepolizumab	Up to 52 weeks	No reliable continuation rule has been identified; initiation criteria best predict benefit.	There was no evidence of a useful continuation rule beyond mepolizumab initiation criteria in SEA.
Silver et al., 2021 [32]	Retrospective database study	USA	327 (117 M, 210 F); mean age 50 years	100 mg mepolizumab SC	12 months	Reduced exacerbations and hospitalizations; decreased OCS use	Mepolizumab reduces exacerbations and OCS use in real-world, life-threatening asthma.
Koistinen et al., 2022 [33]	Retrospective chart review	Finland	51 (21 M, 30 F); mean age 55 years	100 mg mepolizumab SC	Mean 17.8 months	Reduced exacerbations and OCS use; decreased blood eosinophils and FeNO; improved QoL and symptom scores.	Mepolizumab demonstrated effectiveness in real-world SEA in Finland.
Farah et al., 2019 [34]	Prospective cohort study	Australia	20 (12 M, 8 F); mean age 60 years	100 mg mepolizumab SC	6 months	Improved ventilation heterogeneity; correlated with symptom improvement.	Mepolizumab improved small airway function and asthma control in SEA.
González-Pérez et al., 2022 [35]	Observational cohort study	Spain	61 (24 M, 37 F); mean age 46 years	100 mg mepolizumab SC	52 weeks	Improved asthma control and lung function; reduced exacerbations and OCS use.	Mepolizumab was effective and well-tolerated in real-world overlapping eosinophilic-allergic severe asthma.
Nagase et al., 2022 [39]	Retrospective database study	Japan	377 (146 M, 231 F); mean age 62 years	100 mg mepolizumab SC	12 months	Reduced exacerbations and hospitalizations; decreased OCS use; lower exacerbation-related healthcare utilization and costs.	Mepolizumab reduced exacerbations, OCS use, and costs in real-world severe asthma in Japan.
Gupta et al., 2019 [19]	Open-label, non-randomized study	Japan, Poland, UK, USA	36 (25 M, 11 F); mean age 8.6 years	40 or 100 mg mepolizumab SC	12 weeks	Reduced blood eosinophils; well tolerated; higher drug exposure than adults.	Mepolizumab had acceptable PK, PD, and safety in children aged 6–11 years with SEA.
Kroes et al., 2023 [40]	Observational cohort study	10 European countries	912 patients	100 mg mepolizumab SC	12 months	Reduced exacerbations and OCS use; heterogeneity in patient characteristics and treatment patterns.	Mepolizumab was effective in real-world severe asthma across Europe, confirming RCT results using federated analysis of registry data.
Loli-Ausejo et al., 2023 [41]	Retrospective observational study	Spain	44 (13 M, 31 F); median age 57 years	100 mg mepolizumab SC	Median 37 months	Reduced exacerbations and OCS use; improved lung function and asthma control.	Mepolizumab showed sustained long-term effectiveness in real-world SEA.
Kurosawa et al., 2019 [42]	Prospective, open-label study	Japan	32 (16 M, 16 F); median age 63 years	100 mg mepolizumab SC	48 weeks	No exacerbations; increased FEV1 and reduced blood eosinophils.	Mepolizumab demonstrated long-term efficacy and safety in real-world SEA in Japan.
Carpagnano et al., 2019 [43]	Prospective pilot study	Italy	4 (all F); mean age 55 years	100 mg mepolizumab SC	1 year	Reduced exacerbations and eosinophils; improved lung function and asthma control.	Mepolizumab was effective in severe asthma with bronchiectasis, suggesting emerging phenotype responsive to anti-IL-5.

Efficacy Profile of Mepolizumab

The strong effectiveness of mepolizumab in lessening asthma control has been comprehensively demonstrated through pivotal clinical trials, long-term extensions, and real-world studies globally. In randomized placebo-controlled trials like DREAM and MENSA, mepolizumab significantly reduced exacerbations by around 50% and improved lung function and asthma symptom scores over 52 weeks compared to placebo. The long-term benefits have been affirmed through open-label studies like COSMOS, showing sustained reductions in exacerbations and stable lung function for up to 4.5 years. The substantial efficacy has translated to diverse real-world populations beyond controlled study conditions. In observational registries and retrospective analyses conducted across North America, Europe, Asia, and Australia, mepolizumab consistently reduced exacerbations by 50%-90% in less selective patient groups over 6-24 months. Improvements in symptom control, QoL, and lung function were also demonstrated. Though some studies identified subgroups with enhanced responses, such as patients with higher eosinophil counts, most found consistent efficacy regardless of demographics, asthma phenotype, or common comorbidities. Mepolizumab reduced exacerbations and improved asthma control in patients ranging from children to older adults. Overall, mepolizumab's ability to deliver clinically meaningful improvements in exacerbations, symptoms, and lung function has been proven across thousands of patients in RCTs and confirmed in broad real-world populations. The extensive evidence provides unequivocal support for mepolizumab 100 mg SC every four weeks as an efficacious long-term treatment option for SEA (Table 2).

Efficacy of Mepolizumab in Long-Term Management

Several studies have demonstrated that mepolizumab provides sustained benefits with prolonged treatment over years. The open-label COSMOS extension study showed continued exacerbation reductions and stable lung function with mepolizumab treatment for up to 4.5 years. Long-term real-world analyses, including a 2.2-year study by Khurana et al. [12], confirm these findings. Additional RCTs have investigated stopping mepolizumab after long-term treatment. Moore et al. [16] found that patients who discontinued mepolizumab after 52 weeks had more exacerbations and worse asthma control compared to those continuing treatment. This indicates that benefits of mepolizumab persist only with continued usage. Registry studies, such as the one by Korn et al. [21], provide further real-world evidence that mepolizumab maintains stable asthma control and lung function after at least a year of treatment. Similarly, a 37-month study by Loli-Ausejo et al. showed ongoing reductions in exacerbations in clinical practice [41]. Numerous studies have demonstrated the sustained efficacy of mepolizumab over years of treatment, with a loss of benefits when treatment is stopped. Both RCTs and real-world analyses support mepolizumab as an effective long-term maintenance option for patients with SEA. The collective evidence indicates stable effectiveness and acceptable safety for up to 4.5 years (Table 2).

Safety Profile of Mepolizumab

The safety profile of mepolizumab in patients with SEA is well-characterized across numerous clinical trials and real-world studies. In pivotal RCTs like DREAM and MENSA, mepolizumab was well tolerated over 32-52 weeks, with an adverse event profile like placebo. The open-label COSMOS extension study then demonstrated continued favorable safety for up to 4.5 years of treatment, with no new safety signals emerging with the prolonged use. The most reported adverse events were headache, injection site reactions, and back pain, which were mostly mild to moderate in intensity. The excellent safety profile has been held across diverse real-world populations and heterogeneous practice settings. Observational post-marketing registries, retrospective database analyses, and prospective cohort studies conducted globally demonstrated that mepolizumab maintained a safety profile consistent with clinical trials. There were low rates of serious adverse events, immunogenicity, and study withdrawal due to adverse events. Though injection site reactions, headache, and back pain remained most common side effects, these were rarely severe enough to warrant treatment discontinuation. Mepolizumab’s safety has been confirmed in over 6,000 patients through RCTs and thousands more in real-world analyses across ages, backgrounds, and comorbidity profiles. With rare major safety signals emerging over up to 4.5 years of follow-up, the collective evidence demonstrates that mepolizumab has an acceptable safety profile in patients with SEA. Mild injection site reactions and headaches are the most frequently reported side effects, affirming mepolizumab’s favorable benefit-risk profile as a long-term treatment option (Table 2).

Subgroup Analysis and Specific Population Benefits

Some studies have identified patient subgroups deriving enhanced benefits from mepolizumab treatment. Numata et al. found greater improvements in patients with comorbid eosinophilic chronic rhinosinusitis [24]. Similarly, Harvey et al. identified higher baseline blood eosinophils and later asthma onset as predictors of a superior response [37]. However, most studies demonstrate broad efficacy regardless of demographics or clinical characteristics. Ribas et al. showed consistent benefits across eosinophil subgroups [22]. Other analyses found no impact of common comorbidities like nasal polyposis, obesity, or psychiatric disorders on mepolizumab efficacy [28]. Benefits have been demonstrated across ages. Mepolizumab reduced exacerbations and blood eosinophils in children aged 6-11 [18]. Yancey et al. found comparable efficacy and safety in adolescents relative to adults [20]. At the other end of the spectrum, studies in older adults over 60 showed significant reductions in exacerbations and OCS requirements [39]. The excellent tolerability has been held across broad real-world populations beyond controlled trials. Registry analyses and chart reviews have found low rates of serious adverse events, immunogenicity, and treatment discontinuation in heterogeneous groups of severe asthma patients on mepolizumab. No major safety signals have emerged in subgroup analyses. Mepolizumab demonstrated comparable tolerability in children aged 6-11 compared to adolescents and adults. Studies in older adults over 60 found safety profiles similar to younger patients. While some patient factors may predict enhanced responsiveness, mepolizumab demonstrates broad efficacy regardless of demographics, asthma phenotype, or comorbidities. The collective evidence supports mepolizumab as an effective treatment option across ages, from children to older adults with SEA (Table 2).

Discussion

This systematic review consolidates evidence from 32 studies evaluating mepolizumab for SEA. The robust efficacy and favorable safety that RCTs demonstrated have been reproduced globally in real-world populations. Several pivotal trials provided the initial evidence supporting mepolizumab’s efficacy. DREAM and MENSA studies found mepolizumab reduced exacerbations by around 50% and improved symptoms, QoL, and lung function compared to placebo over 52 weeks [8,9]. Subsequent placebo-controlled trials like MUSCA (Mepolizumab Adjunctive Therapy in Subjects With Severe Eosinophilic Asthma) and SIRIUS (Steroid Reduction With Mepolizumab Study) confirmed these benefits in broader patient groups over 24-52 weeks [13,44]. To test efficacy, these RCTs purposefully selected patients with recurrent exacerbations and eosinophilic inflammation.

Open-label extensions of the pioneering trials revealed the durability and stability of effects with prolonged treatment. The COSMOS study demonstrated sustained reductions in exacerbations and stable lung function over 4.5 years of mepolizumab therapy [12]. Other open-label studies like MUSCA showed consistent benefits for up to 3.5 years, with loss of control when mepolizumab was stopped [13,16]. This indicates that the anti-inflammatory effects require ongoing treatment. The real-world evidence provides vital complementary data on effectiveness in heterogeneous environments. Retrospective analyses also found consistent 50%-90% reductions in exacerbations and OCS requirements in less controlled settings [26,32,39]. The benefits have been confirmed across the spectrum of asthma severity and prior medication use [21,23,29]. The longer term real-world studies also found no loss of efficacy over one to three years [12,21,41].

Some studies explored specific subpopulations, including extensive data on children and adolescents. Trials in children aged 6-11 showed mepolizumab effectively reduced exacerbations and eosinophil counts [18,19]. Analyses in adolescents demonstrated comparable safety and efficacy to adults [20]. Studies in the elderly found similar benefits across age groups [39]. Other subanalyses examined patients with comorbidities like chronic rhinosinusitis and found greater improvements with dual airway eosinophilic disease [24]. However, most studies found consistent efficacy across demographics and clinical characteristics [15,22,28].

Regarding safety, mepolizumab exhibited no new or unexpected adverse events with up to 4.5 years of continuous therapy. Most common side effects were minor injection site reactions and headaches [12,18-20,36-43]. Rates of serious adverse events, immunogenicity, and study withdrawal were low across clinical trials and real-world practice. This confirms an acceptable long-term safety profile. Comparative data between biologics remains limited. Indirect analyses found mepolizumab to have similar or superior efficacy to reslizumab, benralizumab, and tezepelumab [14,45]. However, head-to-head trials are needed to determine the relative efficacy of the agents definitively.

The strength of this review is that the analysis encompassed a broad range of study designs, including RCTs, long-term extensions, and real-world observational analyses. This enabled a comprehensive synthesis of efficacy, safety, and effective evidence over short- and long-term timeframes. However, there are some limitations to consider. There was significant heterogeneity across studies in aspects like patient populations, treatment duration, and outcome reporting. Meta-analysis was not feasible due to this heterogeneity. Most real-world analyses were retrospective and subject to the inherent biases of this study design. The observational data, while informative, do not have the rigor of RCTs. There were a limited number of head-to-head comparisons between biologics. The search was also restricted to English language studies, which could exclude some relevant data.

Overall, this extensive evidence base provides robust support for mepolizumab’s clinical utility in SEA. The demonstrated reductions in exacerbations and OCS can potentially modify disease progression and risk of adverse outcomes. Further research should investigate predictive biomarkers to identify optimal patient populations and compare long-term impacts on lung function decline. Additional real-world data is also required for implementation in clinical practice. This review affirms mepolizumab as an invaluable therapeutic option for patients with severe refractory asthma and eosinophilic inflammation.

## Conclusions

This systematic review provides robust evidence supporting the use of mepolizumab as an effective and safe treatment option for patients with SEA. The compiled data from over 32 studies encompassing thousands of patients affirms mepolizumab's ability to reduce exacerbations, OCS requirements, and hospitalizations significantly. The benefits persist with prolonged treatment for up to 4.5 years. The consistent efficacy and acceptable safety profile demonstrated in RCTs have been reproduced in diverse real-world populations globally. While specific patient factors may predict enhanced responsiveness, mepolizumab displays broad clinical utility regardless of demographics or phenotype. Given the demonstrated impact on clinical outcomes and disease progression, mepolizumab represents an invaluable add-on therapy for severe refractory eosinophilic asthma. Further research should optimize patient selection with positioning among emerging biologics. However, the current evidence unequivocally supports mepolizumab as an efficacious therapeutic option for patients whose severe asthma remains uncontrolled on standard treatments.
